# First Year Open Enrollment Findings: Health Insurance Coverage for Asian Americans and the Role of Navigators

**DOI:** 10.1007/s40615-015-0172-1

**Published:** 2015-10-21

**Authors:** Edwin Chandrasekar, Karen E. Kim, Sharon Song, Ranjana Paintal, Michael T. Quinn, Helen Vallina

**Affiliations:** 1 Asian Health Coalition, 180 West Washington Street, Suite 1000, Chicago, IL 60602 USA; 2 Division of the Biological Sciences and Office of Community Engagement and Cancer Disparities, University of Chicago, 5841 South Maryland Avenue, Room S401 MC4080, Chicago, IL 60637 USA

**Keywords:** Accountable Care Act, In-Person Counselors, Asians, Health disparities, National healthcare, Insurance enrollment, Culturally-tailored outreach

## Abstract

The health insurance coverage established by the Patient Protection and Affordable Care Act has created an opportunity to reduce racial/ethnic disparities in healthcare. It is expected that of the 24 million individuals projected to join, nearly one-half will be non-white and one-fourth will speak a language other than English at home. Asian Americans are one of the fastest growing racial/ethnic groups in the USA. The majority are foreign born and experience limited English proficiency. The role of navigators has been shown to increase enrollment rates of public insurance programs. They are trusted for their shared traditions and sense of community. By conducting culturally-targeted outreach, Cambodian, Chinese, Vietnamese, Korean, and Laotian community-based organizations were able to reach individuals for whom the percentage of uninsured is disproportionately high. They enrolled eligible Asians immigrants in coverage despite language barriers and limited health knowledge. Through a collaborative network, a community-level intervention was implemented that was associated with increases in first year marketplace enrollment and greater likelihood of obtaining a primary care physician. Preventable illnesses, lost productivity, and inadequate healthcare are major hardships in immigrant communities that bear similar burdens to society. Bringing primary care to the underserved helps to contain these costs.

## Introduction

One of the aims of the Patient Protection and Affordable Care Act (ACA) has been to expand healthcare coverage. State marketplaces have been created as the mechanism for accessing plans and carriers. Medicaid programs have been restructured so that more Americans with low income now meet eligibility criteria. Individuals and families ineligible for public assistance whose incomes qualify for subsidies receive reduced rates based on a sliding scale (RAND Corporation 2014). Businesses with more than 50 employees have been mandated to offer health insurance (Kaiser Family Foundation 2013).

**Fig. 1:**
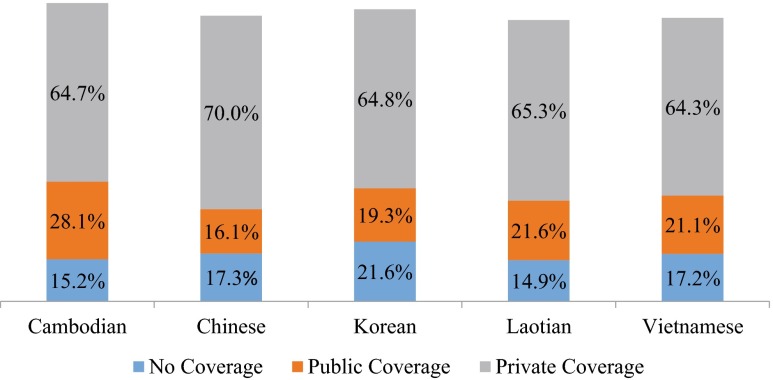
Census reported uninsured Asian Americans in Illinois prior to the ACA

This reform has created the opportunity to reduce racial/ethnic healthcare disparities. By 2019, as many as 24 million individuals are expected to obtain healthcare coverage, of which nearly one-half will be non-white and one-fourth will speak a language other than English at home (Kaiser Family Foundation 2011). There are nearly 18.2 million Asian Americans nationwide, representing 5.6 % of the total population, and estimates suggest that there will be 40 million by 2050 (Le 2014). Asian Americans account for more than one-third of the one million legal immigrants who enter the USA annually (Reinemeyer & Batalova 2007). Approximately 65 % have low incomes and limited English proficiency (U.S. Department of Health and Human Services Office of Minority Health Resource Center 2014). Illinois contains the fifth highest concentration of Asians in the nation and more than 85 % live in the Chicago metropolitan area (United States Census Bureau 2013a). An estimated 1.6 million Illinois residents are uninsured of whom Asian Americans constitute more than 92,000 (15.0 % of the Asian American population; United States Census Bureau 2013b). The uninsured rates by subgroup vary from 15 % of Cambodians and Laotians to 21.6 % of Koreans (U.S. Census Bureau 2012; See Fig. 1). Non-native working adults are more likely to be uninsured than individuals born in the USA (Carrasquillo et al. 2000; Ku and Matani 2001), since fewer immigrants hold jobs with employment benefits such as healthcare coverage (Nguyen et al. 2013).

The ACA incorporated provisions for outreach and enrollment activities designed to help individuals and families choose suitable, affordable plans. The activities included social media campaigns and local partnerships with libraries, churches, state fairs, and sporting events. Reaching out to Asian immigrants at community venues such grocery stores, beauty salons, laundromats, and temples has effectively raised interest in public health issues (Chao et al. 2009; Kim et al.^2009^). One additional initiative that has been effective in promoting healthcare practices in underserved Asian American communities has been the use of navigators. The In-Person Counselor program was established as part of the Illinois state marketplace, known as Get Covered Illinois. In a hepatitis B screening program funded by the Centers for Disease Control and Prevention and the Illinois Department of Public Health, we found that limited English-speaking Asians in the Chicago area who tested positive for the surface antigen were more likely to be referred to follow-up medical care when assisted by a health navigator (Chandrasekar et al. 2015).

Nationally, navigators have been a determining factor of higher enrollment rates in public insurance programs (Stephens & Artiga 2013; Sinaiko et al. 2013). They are known to influence improved health outcomes of underserved communities (Swider 2002). Their success has been attributed to shared ethnicity, religion, cultural traditions, and sense of community (Perez & Martinez 2008). Language concordance is associated with favorable health status (Schachter et al. 2012), and navigators have the ability to communicate knowing first-hand how spoken comprehension of a host country’s language is acquired. The uncertainty of migration is innately understood. Navigators can effectively respond to dissimilar cultural beliefs about health and illness that sometimes perpetuate skepticism toward Western medicine or can create the perception that providers are insensitive. They view the financial constraints hindering access to health insurance for immigrants as situational and surmountable.

Engaging low-income, limited English-proficient Asian immigrant groups has historically produced mixed results for healthcare organizations, providers, and insurers (Sommers & Epstein 2010). At the same time, community-based organizations have been able to gain direct access to the unenrolled eligible cohorts (University of Southern California Division of Community Health 2006). Their collective mission is to help community members overcome langugage and cultural barriers, and attain necessary skills to adjust after migration. Community-based organizations have helped public insurance programs such as the Children’s Health Insurance Program and Medicaid meet enrollment benchmarks because they address the special needs of communities, make public events convenient to participate in, and maintain a stable dependable presence (Cavender et al. 2010). Health workers from community-based organizations have provided outreach and education on such topics as hepatitis B and diabetes and have been viewed as reliable messengers of information (Islam et al. 2014, Taylor et al. 2013; Maxwell et al. 2012). In the hepatitis B screening program mentioned above, the community-based organizations facilitated a reach of nearly 1000 Asian immigrants at health fairs, churches, and other public gatherings in the Chicago area during a one-year period.

In the current study, six community-based organizations participated in Year One Open Enrollment of Get Covered Illinois to educate and assist uninsured members of the Cambodian, Chinese, Vietnamese, Korean, and Laotian communities. They were the Cambodian Association of Illinois, Chinese American Service League, Chinese Mutual Aid Association, Hanul Family Alliance, Korean American Community Services, and Lao American Organization of Elgin. In 2014, their total number of unique clients served was 42,200 (personal communication). Spoken in their native languages, trained bilingual bicultural navigators provided culturally-informed outreach that reflected concordant values and beliefs. They invested time to educate every individual and family, changing negative perceptions and providing the information needed to make appropriate decisions about accessing and utilizing coverage. While applications of best practices and lessons learned are discussed in the context of Illinois and the Chicago metropolitan area, the findings are intended to be applicable to other state programs as well. This study is one of the first to evaluate ACA enrollment at the local level within Asian American communities.

## Methods

The community-based organizations recruited 15 Asian navigators in the Chicago area. Navigators were selected if they had the same language preference, ethnic background, socioeconomic status, and life experiences as community members. Requisite skills included first-hand knowledge of the local culture and available resources, cultural competence, and experience in health education and outreach. The community-based organizations previously recruited facilitators of diabetes and cardiovascular disease self-management workshops and community-wide breast and colorectal cancer screenings, so their selections were made on the basis of the same criteria. The navigators had between 5 to 10 years of prior experience in community health and rated their English language comprehension as “good” on a scale ranging from “poor” to “excellent.” Cultural competency, counseling and mentoring, interpersonal communication skills, motivational interviewing, and chronic disease management were each endorsed by at least one-fourth of the navigators as specific capabilities that they brought to the role.

Proficiency in English was necessary to participate in the mandatory Get Covered Illinois online and workshop trainings. The self-study modules consisted of readings and quizzes and took between 20 and 30 hours to complete (State Reforum 2013). A two-day workshop provided education on such topics as eligibility and enrollment procedures, available health plans, subsidy computations, and privacy and security standards. The required passing rate on the knowledge quizzes was 80 %. Clean fingerprint and background checks were the final requirements determining eligibility for the navigator role.

The purpose of the In-Person Counselor program was to establish hands-on assistance intended to maximize the enrollment of uninsured individuals. Get Covered Illinois trainings emphasized the importance of general outreach principles. They provided technical assistance in using language assistance services, planning for limited English-proficient clients, defining culture, avoiding bias and stereotypes, and managing legal considerations such as non-discrimination. The fundamentals of health insurance coverage, i.e., co-pays, deductibles, open enrollment, utilization review, pre-certification, employer-sponsored insurance programs, etc., were covered; as were enrollment documents, verification notices, reapplication and re-enrollment processes, plan and provider selections, and appeals/adjudication of eligibility determination decisions. In addition, misconceptions were resolved such as about dental and eye care benefits not covered under the healthcare law.

The navigators were taught to avoid calling attention to the magnitude of health problems within a given group (Kreuter et al. 2014). They learned that disparity-framed messages elicited negative emotional reactions and diminished interest in health-protecting actions (Sanders-Thompson et al. 2008). Emphasis was placed on betterment (i.e., “Less time spent waiting in the emergency room is more time to spend with your kids”). Another topic included handling concerns about the affordability and cost of purchasing health insurance. Navigators were trained to focus on the importance of promoting physical well-being and lessening fears associated with illness and inability to pay for care.

Once our program was launched, the navigators employed a variety of strategies, such as door-to-door recruitment and solicitation of word-of-mouth referrals. They linked insurance enrollment activities to existing adult learning, mentoring, and family support programs (i.e., mens groups). In addition to clinics, health fairs, and churches, the navigators worked with their community-based organizations to tap into their neighborhoods as places of opportunity, teaming up with local grocery stores and schools, and conducting outreach events at ethnic festivals and other well-attended gatherings. They canvassed neighborhoods in the days leading up to outreach events introducing themselves to community members personally, and they held multiple enrollment activities simultaneously in collaboration with one another as well as with the small businesses and civic organizations with whom they partnered.

The leveraging of intra-organizational capacity was a defining aspect of our approach. Since language-specific federal and Illinois state materials on the health plans, premium tax credits, fees/penalties, and other topics did not exist for Asian audiences, the staff and navigators of the community-based organizations adapted government-sponsored documents for the health literacy levels of their community members and translated them into the target Asian languages. The resources were then disseminated to the other community-based organizations to create a co-sharing network. Asian immigrants in other countries who did not speak the dominant language were unable to effectively use the computer or Internet (Chiswick & Miller 2007; Ono & Zavodny 2008). In Illinois, the online enrollment form was available in English and Spanish only, so navigators completed the application with community members in their preferred languages in the community-based organization offices where online access was available. Finally, a collaborative community-wide entity came to be formed that allowed the community-based organizations to redirect individuals with conflicting schedules to nearby upcoming workshops, hold joint events, and collectively meet the enrollment benchmarks of the In-Person Counselor program. They used engagement strategies that they knew were culturally appropriate and that could withstand language barriers and limited material resources.

### Participants

Participants in the program were Asian Americans, between the ages of 20 and 65 years, who resided in the USA, and were fluent in English or either Chinese, Khmer, Korean, Laotian, or Vietnamese. We conducted chain-referral sampling, an approach that has been used extensively in nursing and observational research to recruit otherwise difficult to study individuals (Penrod et al. 2003). The method relies on natural social networks and integrates a series of participant-informed referrals into “chain links” so that the resulting sample closely resembles the population of interest. The community-based organizations constituted the first recruitment wave. Selection of the navigators formed the second link. Their involvement with staff of various public gathering places where the enrollment activities were held created the third wave. Establishing visibility in the Asian communities and conducting enrollment activities with community members further lengthened the social network links. As interest in the program broadened to where individual community members became engaged and recruited others to attend the outreach sessions, the referrals chains lengthened yet again—in sum providing the basis of the sampling pool. During the Open Enrollment period, we provided outreach to 3165 community members and successfully enrolled 1000 individuals in health insurance plans.

### Outcome Measures

To evaluate changes in healthcare coverage, we collected data in conjunction with a separate study called the “*Partnership for Healthier Asians*,” led by investigators from the University of Chicago’s Office of Community Engagement and Cancer Disparities and the Asian Health Coalition and funded by the Agency for Healthcare Research and Quality. Responses were part of an individual client survey on evidence-based practices administered to the Cambodian, Chinese, Korean, Laotian, and Vietnamese communities also served by the In-Person Counselor program. The sampling pool was the same as for those who participated in the hepatitis B screening program.

The following demographic variables were collected categorically using pen-and-paper surveys: gender, place of birth, year of migration to the USA, educational background, employment status, household income (in increments of US$10,000), and English language proficiency. Participants reported age as a continuous measurement. Health insurance status before and after Year One Open Enrollment, October 2013 to March 2014, and relationship with a primary care physician were the outcome measures of primary interest (see Table 1 for a detailed description). All variables were formatted as either nominal/binary or ordinal, and responses were forced so “I do not know,” or, “I decline to say,” were not options.

**Table 1:** Individual client survey outcome measures

	Do you have health insurance in 2014?	Did you have health insurance in 2013?	Do you have a primary care physician in 2014?	Did you have a primary care physician in 2013?
YES				
*f*	243	216	257	233
%	77.6 %	65.3 %	75.1 %	68.2 %
NO				
*f*	70	115	85	109
%	22.4 %	34.7 %	24.9 %	31.8 %

### Data Analysis

SPSS Version 21 was used to analyze the data. Participants’ demographic characteristics were summarized using frequencies. We computed *χ*^2^ tests to evaluate whether or not insurance coverage increased when the navigators assisted community members and if selection of a primary care physician likewise changed. We also calculated the correlation between insurance status and endorsement of a primary care physician post outreach using the phi coefficient for binary measures. To evaluate the impact of age, income, and length of time in the USA on current health insurance, backwards stepwise elimination was used in binary logistic regression. All surveys collected from community members were de-identified as part of the data entry step. The project was approved by the University of Chicago’s Institutional Review Board.

## Results

A total of 421 surveys were collected, and 61.3 % were from households with annual incomes of less than US$30,000. The year of arrival to the USA consisted of three primary periods, after the year 2000 (31 %), between 1990 and 1999 (19 %), and before 1990 (45 %). Based on the age distribution of respondents, migration frequently occurred as young adults. College graduation and employment were more commonly endorsed than the other education and vocation categories. Most participants rated their spoken and written English language comprehension as “no better than average.” Table 2 summarizes the characteristics of survey respondents across all community-based organizations and Asian communities.

**Table 2:** Participant profile summary

Survey questions:	Responses options:	Frequency	Valid percent
Gender	Female	204	58.1
	Male	147	41.0
Age	<20 years	5	1.4
	21–30 years	14	4.0
	31–40 years	30	8.5
	41–50 years	44	12.4
	51–60 years	115	32.5
	61–70 years	142	40.1
	>70 years	4	1.1
Ethnicity	Chinese	56	16.3
	Cambodian	47	13.7
	Vietnamese	53	15.4
	Korean	117	34.0
	Laotian	64	18.6
	Other	7	2.0
Year of immigration	Before 1979	31	9.1
	1980–1989	130	38.1
	1990–1999	69	20.2
	2000 and later	111	32.6
Years of education completed	Less than 5 years	53	15.0
	6–9 years	74	20.9
	9–12 years	95	26.8
	>12 years	130	36.7
Current employment status	Employed full-time	135	39.4
	Employed part-time	50	14.6
	Self-employed	19	5.5
	Out of work <1 year	2	0.6
	Out of work >1 year	15	4.4
	Homemaker	20	5.8
	Retired	52	15.2
	Disabled	46	13.4
Household income	Less than US$20,000	161	45.5
	US$20,000–US$30,000	56	15.8
	US$30,001–US $40,000	45	12.7
	US$40,001–US$50,000	26	7.3
	US$50,000–US$60,000	26	7.6
	More than US$60,000	30	8.5
Spoken English language proficiency	Not at all	48	13.6
	Not well	105	29.7
	Average	143	40.4
	Very well	29	8.2
	Excellent	22	6.2
Written English language proficiency	Not at all	55	15.5
	Not well	93	26.3
	Average	132	37.3
	Very well	36	10.2
	Excellent	20	5.6

Participants who did not respond to questions about health insurance coverage or relationship with a primary care physician were excluded from the analyses. While a total of 354 questionnaires were included and contained at least some data on the outcome measures, missing responses occurred most frequently when participants were questioned about healthcare coverage. When asked to indicate current health insurance status, 41 participants (11.6 %) did not respond. Twenty-three (6.5 %) did not answer whether or not they had coverage during the prior year. In contrast, when asked about having a primary care physician, only 12 responses (3.4 %) were missing for the current period and everyone answered the question about the prior year. Overall, the missing data were in proportions that can be considered relatively low (Dong & Peng 2013).

**Fig. 2:**
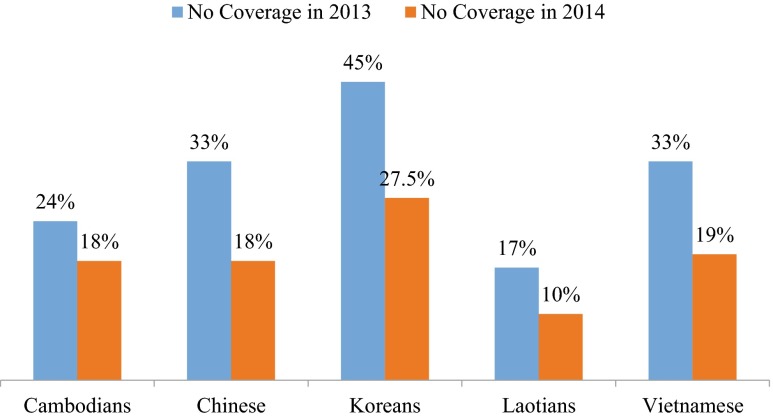
Changes in health insurance coverage of Asian Americans in the study sample

To determine whether or not enrollment was higher after 2014, a chi-square goodness of fit test was calculated with expected insured and uninsured values based on 2013 rates. Prior to Open Enrollment, 34.7 % of community members were not insured. After the navigator assistance program, the percentage of insured Asian community members increased significantly from 65.3 to 77.6 % (*χ*^2^ (1) = 21.02, *p* = 0.000). The proportion of uninsured decreased by 22.4 %, and one reason may have been the involvement of the patient navigators. To evaluate whether or not there was a change in the choosing of a primary care physician, we likewise compared 2014 rates to the prior year. In 2013, 39.7 % of community members did not have established patient–physician relationships. After enrollment, 75.1 % of participants indicated that they did have a primary care physician, which reflected a significant increase over the 60.3 % without a doctor during the prior year (*χ*^2^ (1) = 7.82, *p* = 0.005). The rise of this health seeking behavior in the community was nearly 15 %. Finally, there was a strong positive association between insurance enrollment and having a primary care physician (Φ = 0.673, *p* = 0.000). Individuals with insurance were more likely to have a primary care physician. Figure 2 illustrates the change in insurance status between 2013 and 2014 in our communities.

When evaluating moderating factors on insurance uptake, we found that years since migration and income were significant predictors of current healthcare coverage. The logistic regression model was: CURRENT INSURANCE = 2.98 + .67(YEARS IN USA) + .19(INCOME) + e. See Tables 3 and 4.

**Table 3:** Logistic Regression Model

Predictor:	β	SE β	Wald’s *χ* ^2^ (df = 1)	*p* value	Odds ratio
Constant	2.975	0.557	28.490	0.000	Not applicable
Income	0.191	0.096	3.919	0.048	1.210
Years since immigration	0.665	0.162	16.911	0.000	0.514

**Table 4:** Logistic regression: Overall model evaluation

Overall model evaluation			
Tests:	*χ* ^2^	df	*p* value
Likelihood ratio test	21.019	8	0.007
Score test	28.325	3	0.000
Goodness of fit test	*χ* ^2^	df	*p* value
Hosmer-Lemeshow test	6.559	8	0.585
*R* ^2^-type indices:			
Cox and Snell *R* ^2^ = 0.096			
Nagelkerke *R* ^2^ = 0.146			

Longer length of time since migration was associated with a greater likelihood of having insurance (Wald’s *χ*^2^ (1) = 16.91, *p* < 0.05), as was higher income (Wald’s *χ*^2^ (1) = 3.92, *p* = 0.05) although to a lesser degree. Age did not influence current healthcare coverage and removing it from the model did not improve predictability (Change in −2 Log Likelihood = 2.62, *p* > 0.05). The *R*^2^ goodness of fit measures suggested that between 10 and 15 % of the variability in insurance status was explained by the predictor variables, years in the USA, and income.

When we examined individual subgroups, only the Korean and Chinese participants who were surveyed reflected a significant increase in health care coverage (Korean, *χ*^2^ (1) = 12.81, *p* < 0.05; Chinese, *χ*^2^ (1) = 4.89, *p* < 0.05). The Cambodian, Laotian, and Vietnamese respondents did not report increases that were statistically significant. Descriptively, education beyond high school was highest in the Korean community. Approximately 75 % reported more than 12 years of education as compared to 14–27 % in the other four communities. Income was lowest in the Cambodian and Vietnamese subgroups ranging as high as 72–90 % of respondents reporting incomes ≤US$30,000.

## Discussion

By implementing the Illinois In-Person Counselor program in Asian immigrant neighborhoods, navigators increased the participation of hardest-to-reach families by providing enrollment assistance. Individuals with limited disposable income and English language comprehension participated despite low rates of prior involvement in healthcare services. Some common applications emerged from debriefing meetings that we conducted with the program contributors. Asian navigators who made themselves visible in the communities were viewed as trusted counselors and found it easier to engage individuals and families. We can expect that the face-to-face interactions and the resources created through the formation of the community partnerships allowed this program to have an impact, since the navigators were capable of bridging the language barriers and also language-specific materials for our communities did not previously exist. Improving Internet access for those without it and providing language-concordant hands-on assistance to individuals who were unfamiliar with the online application were also essential. Knowing that language barriers can impede uptake of technology, we found ways to contain their detrimental effects. Additionally, the communities matured in their ability to effect change. Early on, the navigators faced implementation problems, for example, automated warning notices were sent out to enrollees notifying them that their applications had been terminated when in fact they were in process. They reported feeling unprepared to handle the technical errors that intially arose. Yet, at the end of the program, the navigators uniformly reported that working together for the collective well being of their communities and having the opportunity to bring resources to the underserved more than compensated for the challenges and errors.

By instituting national coverage and assisting communities with limited access to health- insurance and knowledge, strides are taken to reduce disparities. Bringing primary care to the underserved contains healthcare costs over the long term. The coverage and relationships with a primary care physician will direct the newly insured to undergo annual wellness exams and regular screenings, remain current with vaccinations, and receive the standards of care that have been shown to promote health and well being. If compliance follows, it will be an important first step toward providing healthcare access to a vulnerable population. Reducing healthcare coverage disparities also limits the misuse of emergency departments as primary care clinics for the uninsured. The costs of preventable illnesses, lost productivity, and inadequate healthcare are major hardships in immigrant communities, and they bear similar burdens to society and the economy.

Prior to implementing the In-Person Counselor program, it was not possible to anticipate the effort that would be required to recruit uninsured individuals for voluntarily enrollment in healthcare coverage. Numerous workshops had to be conducted. Besides planning them ahead of time and oversampling, several impromptu sessions had to be added (especially near the end of the enrollment period) to meet our Get Covered Illinois benchmarks. We saw that without the infrastructure of our communities, it would have been difficult to achieve the flexibility and healthy persuasion of behavior change that led to the favorable outcomes.

In this study, we focused on the Asian community-based organizations that had the capacity to be staffed with navigators. Including a non-intervention comparison group would have strengthened the favorable attributions we made to the them, particularly since a control group likely would have produced lesser or no increase in marketplace participation than the rates we found. Nonetheless, we felt it was important to report on the effectiveness of the navigators in reaching the underserved Asian communities, even if evaluating process outcomes was beyond the scope of the ACA outreach provisions.

In future studies, including other explanatory variables will strengthen the research design. We attributed the changes in insured and uninsured responses to the education and outreach initiatives of the navigators. It is possible that other factors explained the increase in the number of individuals who became insured, including the wish to comply with ACA requirements or avoid the penalty of not enrolling combined with the belief that partaking in national healthcare leads to affordable medical services. In general, we operated under the premise that the navigators and their outreach activities explained the shift in insurance status entirely, but it was possible that the health behavior changes were multifactorial. The results of our logistic regression suggested that characteristics having to do with acculturation and affordability did explain healthcare coverage to some extent, but that there certainly were other explanatory factors not identified here. Examples might include health status prior to Open Enrollment and interactions between race/ethnicity, income, education, and migration period. The Asian communities in our study varied in health insurance and primary care physician uptake, consistently falling below the rates reported for the entire state (compare Figs. 1 and 2). The differences likely reflect the higher concentration of Asian immigrants in the city of Chicago who are without these resources. We only summarize possible additional inferences here because our study was not designed methodologically to capture subgroup differences or control for confounding factors.

Because we used categorical variables, it was not possible to control for household income or the influence of English language skills in our primary analyses. Nonparametric procedures lack the statistical power obtained when analyzing continuous measurements. At the same time, the format we used was necessary to accommodate the limited English proficiency of participants. As discussed above, the individuals who collected the data in the Partnerships for Healthier Asians study obtained the health insurance and primary care physician relationship responses in this project, and the individual client survey was developed from focus group findings a priori to this program.

Few studies have documented first year open enrollment findings of the ACA, and even fewer have examined local data about our specific population. As open enrollment during the second year continues, expanded data will enhance generalizability. What we can conclude at this time is that the use of navigators to implement a community-level, language-specific intervention contributed to an increase in Marketplace enrollment. As expansion of the participant pool remains the primary goal of national healthcare, understanding the specific actions and characteristics that have engaged our underserved community members will be meaningful since there are still many more to enroll.
